# Perspectives on mathematics competitions and their relationship with mathematics education

**DOI:** 10.1007/s11858-022-01404-z

**Published:** 2022-08-08

**Authors:** María Falk de Losada, Peter James Taylor

**Affiliations:** 1 Universidad Antonio Nariño, Bogotá, Colombia; 2 University of Canberra, Canberra, Australia

**Keywords:** Problem-solving, Problem creation, Competitions, Inclusive and by invitation (exclusive), Design science, Mathematical thinking, Argument and proof, Visual thinking, Classroom enrichment, Teacher development, Recreational mathematics, Syllabi and curricula, Mathematics and mathematicians in math education

## Abstract

The nature of the area of mathematical competitions as a design science is considered, historical roots of mathematical problem-solving competitions are traced, the complementary aspects of mathematics as theory building and as problem solving are touched upon in relation to the practice of competitions. Two historical figures, Euler and Erdős, emerge, and the appropriateness of many of Euler’s mathematical ventures are seen as role models for competition mathematics as first put into practice in mathematical competitions towards the end of the nineteenth century. Distinctions and definitions are made, a venture into identifying competition syllabi and the principal types of reasoning employed in solving competition problems is explored, and a description of the many different types of competitions is considered. Interaction between the field of mathematics itself and problem-solving competitions is briefly explored, as are the possibilities that open when competitions and their access to huge amounts of data, both national and international, are taken into account in research belonging to mathematics education. Finally, the range of topics addressed in this special issue of ZDM is covered, along with some possible conclusions relating to the components of the overview.

## Introduction

The subject of the present issue concerns the research and creative activity surrounding mathematics problem-solving competitions and their many facets, objectives and realisations.

The group of authors that has been assembled represents mathematical problem-solving competitions on all continents, as well as international groups bringing together experts from several different continents, both those whose on-going professional work encompasses a global scenario as other ad hoc groups generating totally fresh analyses of various competition initiatives.

### Competitions as an example of design science

Mathematical problem-solving competitions for school (primary, secondary or university) have enjoyed over 125 years of vigorous existence.

Although not necessarily adopted consciously, the conception, structure and evolution of a mathematical problem-solving competition belong to the perspective of mathematics education as a design science in which attention focuses on concrete objects or tools (Lesh & Sriraman, 2010), while the creation of the problems involved requires ingenuity, originality and expertise, and can be considered part art and part science.

Almost all competitions are realized periodically and are based on postulates that underly each different design: the mathematics involved in the problems (informal curriculum), the target age group, the type of problems posed, the type of solution or response, the number of problems included, the time allotted, and so on. The competition may be individual or designed for work in groups, the time frame may be several hours or several weeks, the aim may be encouraging and developing rather than simply discovering mathematical talent, providing teachers with high quality and ever-renewed materials for the classroom, or giving students the opportunity to experience aspects of what it means to do mathematics by confronting situations designed to demand a sustained effort at studying and conquering a problem.

The results of each version of a competition become input for the following one; corrections in the design can be made regarding conditions such as the number of problems versus time or the level of difficulty that the problems are judged to have. The mathematics involved in solving the problems may expand, contract, evolve or simply change according to adjustment or enhancement of the objectives and expectations the designers of the competition may determine or, alternatively, according to the supply at hand of new problems that have been created for the competition.

At another level, competitions are also designed to provide new material of high quality that can be used by the teacher in the classroom, or in extracurricular activities such as math circles, math clubs and math fairs.

Throughout this special issue concentrating on competitions, these aspects will be treated and made clear in a variety of settings and circumstances.

## Some brief reflections on historical roots: ICMI and competitions

In 1969 Hans Freudenthal, then President of ICMI, published an article on mathematical competitions and their history (ICMI Report on Mathematical Contests in Secondary Education (Olympiads) I, Educational Studies in Mathematics) which reveals interest and interaction between the math education community and those involved in the organization of competitions. The reflections he sets forth in the article encompass many of the points that continue to be of interest currently.

1. Types of competitions.
2. Benefits of competitions.
3. Interest in competitions on the part of both mathematicians and school teachers.

He also repeatedly touches on the “pipeline” and the formation of the mathematical community as related to problem-solving competitions.

Freudenthal looks closely at a variety of competitions and notes the different aims behind the different events, singling out, for example, a local competition in the San Francisco area which he deemed important and strategic enough to detail.

“A St. Mary's College (California) program [85] aims at encouraging and developing rather than discovering mathematical talent. …The program provides high-school and junior high-school students with an opportunity to solve problems of some difficulty not just once a year at the time of a mathematics contest but throughout the year. The aims envisaged by the sponsors are: (1) To arouse mathematical interest, especially in key areas of problem-solving; (2) To discover mathematical talent that might otherwise remain hidden; (3) To help students keep widely separated parts of mathematics freshly in mind; (4) To direct abler students to advanced techniques, useful notations, and to habits of generalization and abstraction; (5) To upgrade performance in homework, examinations, and contests; (6) To help teachers continue developing their own skills.” (Freudenthal, 1969 p91–92) This competition is related to the ongoing Julia Robinson Mathematics Festival.

Many of the reflections Freudenthal shares continue to find echo. For example, “prize winners have a good chance to become clever mathematicians but not all gifted children are good in competitions, and failures may discourage them. This is a disadvantage, which, however, is outweighed by the pleasure of problem solving. Whoever discovers its beauty enjoys it better and better.” (Freudenthal, 1969, p. 100).

With regard to problem creation, we find our author citing a Hungarian problem from 1940 [Supposing that among certain objects there are some of different colour and some of different shape, prove that there are two among these objects that differ both in colour and in shape.]. He underlines the fact that a problem can be very simple but, in being unusual for its time, it tests inventiveness rather than rote, and notes that it is not easy to find problems that are both new and not forbidding.

He finally asserts that “Contests can profitably supplement teaching by raising interest, by stimulating problem solving, by providing teachers with fresh problems, and they can even indicate possibilities to improve classroom work.” (Freudenthal, 1969, p. 100).

Of course, Freudenthal attributes these developments to their origin in Hungary in 1894 when the first national competition was begun as part of the celebration of 1000 years of the presence of the Hungarian people in the land they continue to occupy. An outstanding physicist, Loránd Eötvös was then Hungarian Minister of Education and his project to celebrate and promote national scientific excellence was thus auspiciously launched. The story of how this spawned the founding, nourishing and maturing of a Hungarian mathematics community that stood out internationally became an example to be emulated (Kenderov, 2022).

Mathematical competitions and challenges as studied in this special number of ZDM continue to thrive outside of the formal school environment. As we will see in the following section, they provide a common ground for mathematicians and mathematics educators to collaborate in exploring original problems, offering opportunities to work on them, as well as opening young minds to the depth, geniality and intricacies of basic elementary mathematics and inviting them to develop the reasoning required to solve them.

We offer a brief discussion on types of competition below under the heading of definitions.

## Mathematicians and mathematics educators come together

Many mathematicians have devoted a significant part of their lifetime work to mathematics education. Mathematics education is a field whose first leaders were virtually all educated as mathematicians. Many from these first generations are still actively leading the field. This relationship was built into the structure of ICMI as a standing committee of IMU and the manner in which the presidents of ICMI were elected. Highly talented and visible mathematicians such as Felix Klein, Marshall Stone and Hyman Bass contributed vastly to the field of mathematics education while presiding over ICMI.

Mathematicians and mathematics educators continue to find a field for fertile collaboration in mathematical problem-solving competitions. Perhaps it is the only area of mathematics education where mathematicians actively involved in research on the frontiers of the field also formulate stimulating, highly challenging and frequently beautiful problems for the most exacting of the problem-solving competitions and their offshoots such as the summer research camps for high school students associated with the Tournament of Towns.

## Some historical roots

### Mathematics and challenge

Challenges and competitions are rooted deeply in mathematical history and continue to play a fundamental role in its development over time. We have written of the profound changes in the nature of the mathematics being done as result of a competition launched in the twelfth century which led to some original thinking in the mind of Leonardo de Pisa (Fibonacci), initiating the transition from geometrically based to algebraically based mathematics in Europe, and introducing precociously arguments tantamount to assuming the intermediate value theorem for polynomial functions. (Falk de Losada, 2017).

The renowned intrigue, scandals and plagiarism in Renaissance Italy involving figures such as Cardano and Tartaglia and centring on finding formulas for solving the general cubic and quartic equations, occurred in the context of public challenges and competition.

Beginning with the modern scientific revolution, European mathematics in general advanced through correspondence, informal academic gatherings, ad hoc groups, and scientific societies, among other milieus. Mathematicians directly exchanged problems they were dealing with, and both jointly and separately set out to solve them. With the exception of England, mathematics in Europe developed outside of the university environment up to the grand transformation of the university commencing in the first decades of the nineteenth century (Kline, 1972). In a parallel fashion the nineteenth century saw mathematical activity and research move solidly into the university.

These changes set the stage for the development of a separate profession, that of mathematician. A distinction between pure and applied mathematics began to appear in the minds of thinkers such as Gauss and Bolzano (Falk de Losada, 2012). Mathematical research as an end in itself and independent of its applications “led to the establishment of new professorships in mathematics at universities throughout Europe, to new periodicals dedicated to pure mathematics, and to professional societies for mathematicians. All of these things were virtually non-existent only a century before.” (https://www.encyclopedia.com/science/encyclopedias-almanacs-transcripts-and-maps/overview-mathematics-1800-1899).

Concomitantly, the explosion of mathematical knowledge led to increased specialization among mathematicians so powerful that by the end of the nineteenth century it was generally accepted that Henri Poincaré was the last mathematician to know all the mathematics being done in his time. Specialization also came to imply that the open problems being researched required several years of study (generally at university) in order for mathematicians to be able to begin to tackle them.

In this line of mathematical development, the presence of challenge in the form of lists of problems to be researched and solved can be seen throughout the twentieth century in the interventions of Hilbert, Erdős, Graham, and the Clay Institute. Constance Reid (Reid, 1970) tells us that Hilbert launched his famous list of unsolved problems to show that there was still much work to be done in mathematics and to attract promising young thinkers to the field. Erdős, Graham, the Clay Institute and others have followed in his footsteps, adding the lure of cash prizes perhaps following the tradition of awards recognizing stellar research work, such as the Nobel prizes. Problem-solving challenges remain an outstanding feature of research in mathematics in the twenty-first century.

### Mathematical recreations for the general public and original problems in school mathematics

The second half of the nineteenth century also saw a resurgence of interest in what has been called recreational mathematics. Recreational mathematics is another root of the problems created and considered in challenges and competitions on the school level. It also forms part of the scenario for the emergence of mathematical competitions. For school teachers of mathematics, and for a new generation of mathematicians and aficionados, it became apparent -given the newfound, highly specialized nature of problems on the frontiers of mathematics- that new and challenging problems accessible to students and a public intrigued by puzzles and problems requiring only elementary mathematics were urgently needed.

We have advanced the idea that the renewed interest in recreational mathematics and the beginning of competitions of problem-solving on the school level are a natural consequence of the increasing abstraction, formalization and specialization of mathematics in the nineteenth century that essentially made unsolved problems on the frontiers of mathematical research unreachable for young students (Falk de Losada, 2017), leading up to the proposing of intriguing, genuine and singular problems that young minds could grasp and work on and also giving students a taste of what it means to do mathematics as a means of developing their creativity, and attracting problem-solvers as well as theory-builders to mathematics (Falk de Losada, 2017).

Here begins the line of development in mathematics education generally associated with problem solving competitions.

### Problem-solving and theory building

Many of the historical origins of recent mathematical challenges and competitions, as well as their general flavour, can be found in the work done by Leonard Euler, immersed in a tradition furthered greatly in the twentieth century and personified by Paul Erdős (Falk de Losada, 2017). Timothy Gowers (IMO Gold medalist, 1981 as well as Fields Medalist) has written on several occasions of the dual nature of doing mathematics, on the one hand theory building and on the other problem solving, and of the fact that all mathematicians participate in both these activities but tend to privilege one of them above the other as their fundamental motivating force. (Gowers, 2000, 2008).

### Erdős echoes Euler

Paul Erdős was a singular figure of twentieth century mathematics, educated at home as a young child and deeply involved in mathematical problem-solving competitions as a teenager. His exceedingly non-standard life as a mathematician has been amply discussed elsewhere (see, for example, Bollobás, 1997). His focus on problem posing and problem solving is highly noteworthy, and his accomplishments legendary. Nevertheless, his achievements echo in some way those of a figure of the eighteenth century whose mathematical interests and accomplishments can be seen as precursors of both the competition and recreational mathematics that emerged in the nineteenth century. We are speaking, of course, of Leonard Euler.

From the mind of Euler graph theory (exemplified by the problem of the bridges of Königsberg), Latin squares (the basis for today’s sudoku puzzles), number theory (generalizing several of Fermat’s achievements), the formula for polyhedra involving vertices, edges and faces, solutions to problems left by Euclid (the converse of his theorem involving perfect numbers), by Fermat (the proof of the case of Fermat’s last theorem when *n* = 3, 4), and many others emerged. His correspondents included Christian Goldbach famous for his still unsolved conjecture (the mathematical community continues to evaluate the solution given to Goldbach’s weak conjecture by Harald Helfgott, a Peruvian mathematician whose mathematical career began with his participation in the Iberoamerican Math Olympiad (Fauring et al., 2022, this issue). Working outside of the university environment, Euler, in addition to his contributions to mathematics proper, was a consummate problem solver and his achievements belong to a great many of the areas of mathematics contained in the different competition syllabi as they have evolved in the intervening centuries of development in mathematics.

## Some definitions

Thus a brief history of competitions of interest to this overview starts in the modern era in Hungary, in 1894, where we see the start of their national Eötvös Competition, which also coincided with the start of the student journal KöMal. It is important that competition problems can be discussed, so most competition organisers will have ways of direct contact with students as well as teachers, and journals are just one of these ways.

Such competitions as the Hungarian one would normally be for the more talented student, who by personal interest, extra training or natural ability can handle more challenging tasks, as noted by Freudenthal, and would normally only be via invitation, in this case by the teacher, and so would often be called exclusive.

The next generation of competitions would be the Olympiads of Moscow and Leningrad, in the USSR during the 1930s. These events would definitely be described as exclusive.

After World War II competitions for all students were started in the USA, then Canada, Australia, South Africa, the United Kingdom, and in the 1990s the Kangaroo competition, which was later to be available internationally, was founded in France. These events were designed for students of all standards, giving an assessment external to a student’s school, without the pressure of worrying about consequences of performance. These events became massive and were not only easy for teachers to run, but they often also helped to identify a student who had a hidden talent, as there are students of outstanding promise who do not perform so well in the school environment. These competitions, open to all students and designed to be familiar from classroom experience, are called inclusive.

Since World War II there has also been a rapid growth in exclusive competitions, some on national scales, some on regional scales and ultimately there is the International Mathematical Olympiad (IMO), in which teams of the top six students from each participating country gather in one location, giving them also the opportunity to meet counterparts from other countries.

Whereas the dichotomy suggested above of inclusive (open for students to enter) and exclusive (only by invitation by teacher or organisation) appears clear-cut there are many variations.

We note that there is a paper in this issue by McAvaney referring to an Australian Competition known as the Challenge. This event is technically open in many schools in that students can enter of their own accord, but the competition is designed to allow interested and talented students to extend and enrich their mathematical knowledge beyond the classroom experience. This is usually done by guiding the student in steps starting with a familiar idea and then extending situations. So, whereas the event is arguably inclusive it has the characteristics of helping the student to develop with an aim towards more exclusive situations.

Highly noteworthy is the Tournament of Towns, a competition that is administered in Moscow but is available in other countries and has two components, known as O level and A level (students may enter either or both). Students may in some places initiate their own entries, but it has the hallmarks of being “exclusive”. The O level paper is somewhat gentler and can have problems close to classroom level, although normally putting somewhat higher demands on students, whereas the A level can be particularly difficult, some problems being more difficult than in IMO.

So, whereas people associated with competitions sometimes use the terms inclusive and exclusive, these are general terms and there is a very wide range of types of competitions held in various places that can fall into these categories.

All countries, especially those that participate in IMO, will have a national Olympiad, which will be a competition used in the selection of the national teams (not normally done by a single examination). There are also, around the world, a number of regional Olympiads, usually held in several countries with geographic proximity or a shared language. One regional Olympiad is the well-known Ibero-American Olympiad, which originated in Colombia and whose participants come from Spain, Portugal and the Latin American countries. This Olympiad is referred to in this edition in a paper by Fauring et al. (2022), this issue. There are a number of other similar competitions, such as the Asian Pacific Olympiad, the Balkan Mathematical Olympiad and the Baltic Way Olympiad to name a few.

These regional Olympiads are generally regarded as fitting between national Olympiads and IMO in difficulty, and frequently used as selection exams for national teams. These regional events, as well as national Olympiads, would be classed as exclusive, with students in Olympiad training programs invited by their national committees.

Several mathematics competitions (including the Asian Pacific Math Olympiad, the Tournament of Towns, or the Olimpiada de Mayo organized by Argentina for Iberoamerican students under 15 years of age) share a design which encourages wide participation since the “team” of each school or country is determined a posteriori, based on the results achieved in the competition itself and not on previous performance.

There are other types of competition held in different countries, not in general run by organisations such as national committees, which are designed to be held in various ways that cannot easily be classified as inclusive or exclusive. Each design is intended to respond to specific aims and to play a specific role in eliciting, nurturing and developing students’ mathematical thinking.

Regarding types of competition, events like the Hungarian and national and international competitions are traditional, with a pen and paper exam style, and are usually hand-marked.

One notable exception among the earlier-established events was the Leningrad (now St. Petersburg) Olympiad, which was always set in the traditional way but answered verbally in an interview, and then allotted marks in a traditional way.

The large inclusive competitions, which are too big to mark by hand, were traditionally set in normal classrooms and answered with pen and paper on mark sense sheets which can then be read for computer marking. But in recent years, with rapid technical development, many of these have been available also online. In 2020, due to the pandemic, in many countries it was fortunate that on-line methods had been developed, as these were the only means possible.

There are many other variants in which smaller, even inclusive (for school classes) competitions, for which different types exist. Some are mathematics days, in which schools in neighbouring towns might send a small team to compete in the one location with different styles during the day, and which can contain variations such as mathematics relays.

There are also the research events, such as the one associated with the Tournament of Towns, that are designed and organized as summer camps.

We and many of the authors of articles in this special number have been involved in many other different types of challenging situations, which were covered in ICMI Study 16 (Barbeau and Taylor, 2009), but which we regard as beyond the scope of this issue.

### Technical advances

As mentioned above, new technologies are being used in large, inclusive competitions, a development which was quite timely, as it has given organisers already a solution for holding such competitions during the COVID-19 pandemic. This is discussed in more detail in Kenderov (2022).

In fact, during the pandemic students and leaders have not been able to travel for Olympiads, and these events have proceeded using the development of communication methods such as Zoom and Skype with much success. One does assume though that for these events, organisers will prefer to come together for the Olympiads when restrictions on international travel are lifted.

## University competitions

There are a variety of university level competitions in different regions and countries of the world. The three described below, based on webpage information, illustrate the array of designs that can be found.

### The William Lowell Putnam competition

The competition was founded in 1927 by Elizabeth Lowell Putnam in memory of her husband William Lowell Putnam, who was an advocate of intercollegiate intellectual competition. The competition has been offered annually since 1938 and is administered by the Mathematical Association of America. The Putnam Competition covers a range of material in undergraduate mathematics, including elementary concepts from linear algebra, modern algebra, analysis and number theory.

The Putnam began as a competition between mathematics departments at colleges and universities. Now the competition has grown to be perhaps the leading university-level mathematics examination in the world. Although participants work independently on the problems, there is a team aspect of the competition as well. Institutions are ranked according to the sum of the positions occupied by the three participants designated officially as their representatives or team. Prizes are awarded to the participants with the highest scores and to the departments of mathematics of the five institutions the sum of whose top three scores is greatest (https://www.maa.org/math-competitions/putnam-competition).

The Putnam is held on each university’s campus in the month of December, however the 2020 version was postponed due to the Covid pandemic. We note also that Harvard and to a slightly lesser degree MIT have been the most successful over the years; the event has been won by a number of other universities as well (https://www.maa.org/math-competitions/putnam-competition).

### University College London International Mathematics Competition—IMC-founded 1994

University College London has been involved in the organisation of the IMC and Professor John Jayne has served as the President from its beginning in 1994. In a design somewhat similar to the IMO, the IMC runs over five or six days during which the competitors sit two five-hour examinations, each with five questions chosen by a panel in association with representatives from the participating universities. Problems are from the fields of Algebra, Analysis (Real and Complex), Combinatorics and Geometry.

From 1996 to 1999 the IMC was one of the activities of the Structural Joint European Union TEMPUS project, entitled "Modular Education in Mathematics and Informatics", which was a European Union project in Bulgaria at the time, aimed at bringing Bulgaria's university mathematics and computing degree programs into line with those in the European Union in preparation for Bulgaria's entry into that organization. The IMC has expanded to include university teams from other latitudes, counting recently with about 80 universities among its participants.

Usually meeting in Bulgaria, the IMC was held virtually in 2020 (https://www.imc-math.org.uk/).

### Competencia Iberoamericana Interuniversitaria de Matemáticas—CIIM—or Iberoamerican Interuniversity Mathematics Competition, founded 2009

As its webpage tells us (http://ciim.uan.edu.co), the Iberoamerican Interuniversity Mathematics Competition came into being with the idea of incentivating the study of mathematics and academic excellence in the Iberoamerican university community, by bettering scientific capacities through motivation and promotion of international competitivity and thus contributing to social, cultural and economic development in the Iberoamerican countries. The Interuniversity competition is designed for undergraduate students familiar with basic concepts of number theory, geometry, combinatorics, calculus and algebra, competing on a single level.

The CIIM has been organized by the Universidad Antonio Nariño (which also organizes the Olimpiadas Colombianas de Matemáticas) in different cities in Colombia, by the Instituto Militar de Engenharia (IME) and the Instituto de Matemática Pura e Aplicada (IMPA) with support from the Olimpíada Brasileira de Matemática (OBM), and is currently organized by the Universidad de Guanajuato, México.

The Competition was held virtually in 2020.

### A sample of other university competitions

There are many other university level competitions in the world, each with its academic design and format. In order to appreciate the variety, we mention the following three.

1. Indiana's Friendly Mathematics Competition founded at Wabash College in 1965 and known today as the Indiana College Mathematics Competition, emphasises teamwork. Students within a team cooperate and the teams submit one solution per question. Each team determines how to manage its work and time: Some teams are truly collaborative, whereas others carry out a divide and conquer strategy, with different members working on different problems.
  The number of problems varies from six to eight per year, no calculators are allowed, and each year's problems include some problems everyone should be able to do, along with those that challenge and allow for distinguishing among the problem solvers (https://en.wikipedia.org/wiki/The_Indiana_College_Mathematics_Competition) founded
2. Hungary's Miklós Schwitzer Competition, which is a high-level rivalry among research-oriented students at the university level, who are given 10 days to solve 11 problems. It is an open-book competition. (http://www.math.u-szeged.hu/~mmaroti/schweitzer/)
3. COMAP (Consortium for Mathematics and Its Application) organises the Mathematical Competition in Modelling (MCM) which is a competition between teams, each consisting of at most 3 students, who are to use mathematical modelling in providing solutions to real life problems and are also allowed the use of inanimate sources. They have a choice among 6 problems and are given an extended week-end (usually, from Thursday to Monday) to write up their solutions. (https://www.mathworks.com/academia/student-competitions/mathworks-math-modeling-challenge.html)

## Competitions and career choice

A paper by Taylor (2017) investigated how students were encouraged by their competition experience to appreciate mathematics beyond their classroom milieu. To quote the responses of three such students in a survey on the Australian competitions,

- “A source of pride—we were immensely competitive in a good-natured way at school and there were three or four students in my year who won AMC medals in various years. We still get together every year to do the... AMC competition paper over dinner (our 15th year this year).”
- “Selection into the Mathematical Olympiad training programme, with many flow on benefits, including: learning much more mathematics and at a higher level, meeting like-minded people many of whom are now good friends, getting encouragement to continue with mathematics.”
- “I think (it) got me an invitation to participate in the Tournament of the Towns—which in turn meant regular exposure to (a) more challenging mathematics, and (b) other extremely talented students. I gained a great deal from this program. At school, I got somewhat embarrassed by the fuss and teacher pride, but on the other hand my teachers were happy to let me do other things in class once I finished class work.”

In this paper the future careers of Olympians of Germany and Australia were also traced, looking at the data in Engel et al. (2009) and Taylor (2013). It was found that of those in German teams (East Germany, West Germany and later unified teams) there are 253 students identified, 45 of whom had unknown later careers. Of those known, 148 obtained mathematics-based doctorates and a further 50 studied mathematics and pursued mathematics-based careers. Members of Australian teams up to 2017 numbered 118, and of those at least 99 of them were found to have pursued mathematics-related careers, with the future career of 5 students unknown.

Taylor and McAvaney (2020) noted a correlation between Fields Medal winners and IMO Gold Medallists and listed the following mathematicians in both categories.

- Vladimir Drinfeld, USSR/Ukraine, IMO Gold Medal 1969, Fields Medal 1990.
- Jean Christophe Yoccoz, France, IMO Gold Medal 1974, Fields Medal 1994.
- Richard Borcherds, UK, IMO Gold Medal 1978, Fields Medal 1998.
- (Sir) Timothy Gowers, UK, IMO Gold Medal 1981, Fields Medal 1998.
- Grigori Perelman, USSR/Russia, IMO Gold Medal 1982, Fields Medal 2006 (declined).
- Stanislav Smirnov, USSR/Russia, IMO Gold Medal 1986, 1987, Fields Medal 2010.
- Terence Tao, Australia, IMO Gold Medal 1988, Fields Medal 2006.
- Ngô Bao Châu, Vietnam/France, IMO Gold Medal 1988, 1989, Fields Medal 2010.
- Maryam Mirzakhani, Iran, IMO Gold Medal 1994, 1995, Fields Medal 2014.
- Artur Avila, Brazil/France, IMO Gold Medal 1995, Fields Medal 2014.
- There are other Fields Medallists who participated at IMO but with lesser outcomes.

### Pipeline project

In the first decade of the XXIst century, IMU conducted a study under the aegis of ICMI about issues associated with the supply and demand for mathematics students and personnel in educational institutions and the workplace. Present in Freudenthal’s survey of competitions fifty years ago, it is clear that the mathematical community has looked to mathematical problem-solving competitions to increase the supply of candidates who will take up mathematics as a profession. For example, in the study that formed the basis of a talk at the WFNMC Congress of July 2018 in Graz, Austria (Falk de Losada, 2020), we showed the increase in the number of university programs in pure mathematics in Colombia parallel to the founding and growth of the Colombian Mathematics Olympiads. Before the Olympiad, the existence of professional programs in mathematics was not even a generally known fact in Colombia.

The article by He, Ling and Xiong (2021) in this special number investigates, among other things, the relationship of competition experience to career choice in China.

## Country roundup

The development of competitions, since the first recorded one of the modern era in Hungary in 1894, is varied. In this special edition the editors have obtained a special paper on China, written by He et al. (2022), an article from Fauring, Gaspar y Losada about competitions in Iberoamerica (Fauring et al., 2022), and a review of competitions in Africa as a continent, written by Baker et al. (2022). Here we will review the development and current phases in certain other countries of significance.

### Russia

As previously stated, the first notable competitions in Russia were the Moscow and Leningrad Olympiads in the 1930s. The Moscow Olympiad is a very orthodox type of exam for the most gifted students. The Leningrad Olympiad was unusual in that students gave verbal rather than written solutions.

A national USSR Olympiad on top of these followed, together with national Olympiads in other Soviet Bloc countries.

These led, particularly due to the Romanian mathematician Tiberiu Roman, to the first International Mathematical Olympiad (IMO) held just among the Soviet bloc countries, in Romania in 1959. Eventually Western countries were able to join, and the IMO had become truly international by the 1980s.

One of the interesting developments by the 1980s was the establishment of “circles”. Circles were informal groups of students mentored by local teachers committed to continuing the history of high quality of mathematics in a way which schools, which catered for the whole population, could not do.

These circles met regularly under the hosting of their mentor, who developed student knowledge with well-designed plans. Students in these circles became those who would be the winners of the National Olympiads.

One of the most notable mentors was Sergey Rukshin, a teacher in Leningrad, and one of his most notable students was Grigor Perelman, later winner of an IMO Gold Medal for the USSR and Fields Medal, which he declined. There were many other very successful students who had been in a circle environment. Circles also developed in other countries, including countries in the West.

Also in the 1980s, a different type of competition started. This was the Tournament of Towns, in which towns or cities from Russia, and originally also Riga, entered teams, the number of students whose score would be taken into account being proportional to the population. This developed through some other members of the Soviet Bloc and in 1988 Canberra became the first Western city to enter. The Tournament became affiliated with the Soviet (later Russian) Academy of Sciences.

Together with the competition referred to above, between cities, the Tournament also established a unique summer conference, to which the best students are invited to experience research, by being provided with problems, some unsolved, and giving the students a few days to make progress, whether individually or in collaboration.

Russia today has many more competitions and challenges and is a rich source of innovation in setting original problems.

### USA

The real history of competitions in the USA coincides with the post-war arrival of such people as George Polya at Stanford, with his experience of the value of the enrichment of mathematics learning in Hungary.

Without going into a detailed history, the main formal national competitions resulted in management under the auspices of the Mathematical Association of America (MAA), which founded the large inclusive American Mathematics Competition (AMC) about 1950. Later they founded the USA Mathematical Olympiad (USAMO) which was eventually used to identify students when USA entered the IMO. In 1982 the MAA also founded an intermediate event, the American Invitational Mathematics Examination (AIME) which provided a pathway between AMC and USAMO, with entry limited to invitation based on AMC results, and then USAMO with participation on invitation based on AIME performance.

Another early event of real significance was the ARML, earlier Atlantic Region Mathematics League under leadership of Al Kalfus, later the word “Atlantic” replaced by “American”. This was a live event in which school teams from a wide geographic area would travel to a common location to compete.

Another was the USA (also International) Mathematics Talent Search, under George Berzsenyi.

It is not possible to go further without risking the overlooking of good events but there is now a complex network of local and regional competitions in the USA.

### Canada

Soon after the USA AMC, the Canadians started a similar competition based at the University of Waterloo, a new and strong university with a scientific and engineering emphasis, which used their competition as a method of awarding scholarships to the best students. This event was notable also for having a strong support program for teachers.

### Australia

In 1973 Peter O’Halloran returned to what is now the University of Canberra after spending a year’s study leave at the University of Waterloo. He arrived after becoming familiar with both the Canadian Competition and the AMC and had a vision for a model in Australia, which would not promote a particular University nor be subject to a higher professional society such as the MAA. He built strong support from some colleagues and by 1976 had begun a competition in Canberra, which became so popular with teachers it quickly spread as the Australian Mathematics Competition (also AMC) across Australia and in fact to much of the Pacific and Southeast Asia, including such countries or regions as Singapore, Malaysia, Brunei, Philippines, Indonesia, Taiwan and Hong Kong.

The organisation developed as a training place for Australia’s participation in IMO, actually hosted IMO in 1988, and developed an intermediate program called the Challenge, which is discussed by Kevin McAvaney (2022). In 1992 all of these events were placed under a single organisation known as the Australian Mathematics Trust (AMT), a not for product organisation, financially independent under the Trusteeship of the University of Canberra.

### France

By 1992 the French had noticed the popularity of the Australian model and developed their own equivalent, and in honour of their inspiration named it the Kangourou. The Kangourou became exceptionally popular in France and by 1996 had spread through Europe, with a model very similar to the Australian AMC. The Kangourou now stands internationally as one of the largest events of its type, spread right across the world.

### United Kingdom

The UK also set up a national organisation, based on the Australian AMT but designed for local conditions, and which has become a very strong organisation for mathematical competitions.

### Others

There are now similar organisations and events in other countries, particularly national Olympiad committees in most countries, for identifying IMO teams, but we have described the main developments, particularly of school-based inclusive events, which have become of great use for teachers, not only in gaining an extra insight into the talents of their students, but also providing a pathway for the more talented students to enrich their mathematical knowledge beyond what they can learn in the classroom.

## Competition syllabi

From the Hungarian Competition on, many competitions have an unwritten syllabus with an emphasis on structural ideas rather than calculation.

One thing which surprises mathematicians is that the main inclusive and exclusive pre-university competitions never have calculus. If there is a calculus solution to a problem, then problem setters would normally ensure that there is a better (i.e. more elegant and succinct) non-calculus solution.

At the highest level for secondary school—the IMO—there has not been a defined syllabus; problems are contributed by participating countries, screened and subject to preselection by a problem committee appointed by the host country, and offered to the jury for final selection on a shortlist, and traditionally come under one of the four categories of algebra, combinatorics, geometry or number theory. The jury, which comprises the country team leaders meeting in isolation from their students, will choose the paper from the shortlist and make subjective judgments on the paper, taking into account the history of the event.

Exclusive competitions will generally choose problems under the same headings. However, one of the largest international exclusive events, the Russian-based Tournament of Towns, tends to be more flexible in their choices than is the IMO.

The paper in this issue by Nieto and Sanchez (2022) discusses competition syllabi and their possible relation to school curricula with a particular emphasis on exclusive events.

At the very school-based end of inclusive competitions, the problems committee will generally consider the school system as the syllabus, while still choosing questions involving structure rather than calculation. Taylor (2015, 2017) attempted a classification of topics found in a variety of inclusive and exclusive events. Many of these topics are not in the usual school syllabus but normally students can easily extend their knowledge to include these.

Taylor (2015, 2017) gives also a typical example from a competition for each topic, which will not be done again here. But the topics listed include all the Euclidean geometry topics to be found in a school syllabus, mechanics, a lot of discrete mathematics (counting methods, discrete optimisation, graph theory, Diophantine equations, etc.) and a lot of logic and methods of proof. Other methods include inverse thinking, looking for an invariant, colouring and probability. Some of these are not discussed in many classrooms but can be set in frameworks to which a talented student can adapt.

### An illustrative example

Most of the topics above will be familiar to the reader. But some competitions, in particular the Russian based Tournament of Towns, will have associated journals (in the Russian case the Journal *Kvant*), which discuss new ideas which may sometimes be used in the competition papers. One example is the method of the moving parallel. This is related also to ideas put forward on dissection of polygons by Kasimatis (1989) and Galvin (1990).

The following problem was set in this competition earlier, in 1983, and illustrates this method.

### Problem

A regular 4* k*-gon is cut into parallelograms. Prove that among these there are at least *k* rectangles.

### Comment

This problem does not seem to be solvable by methods such as Euclidean methods found in the classroom, but in fact the following solution by Andy Liu illustrates this method and is not difficult.

### Solution

Let the regular 4* k*-gon be dissected into parallelograms. Let *x*_1_ and *x*_2_ be a pair of opposite sides. The set of all parallelograms with one side parallel to *x*_1_, starts from *x*_1_ and eventually reaches *x*_2_, possibly subdividing into several streams. The diagram illustrates the case of a regular octagon.
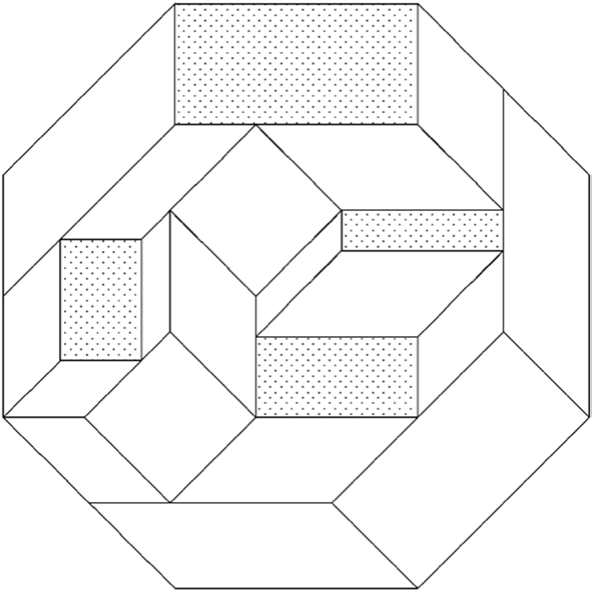


Since the regular polygon has 4* k* sides, there is a pair of opposite sides *y*_1_ and *y*_2_ perpendicular to *x*_1_ and *x*_2_. The set of parallelograms with one side parallel to *y*_1_ starts from *y*_1_ and eventually reaches *y*_2_, again possibly subdividing into several streams.

Now these two sets of parallelograms must cross each other. This is only possible at parallelograms with one pair of opposite sides parallel to *x*_1_ and the other to *y*_1_.

Since *x*_1_ and *y*_1_ are perpendicular, this parallelogram is actually a rectangle (due to subdividing into several streams, four such rectangles based on *x*_1_, *x*_2_, *y*_1_, *y*_2_ in the diagram, exist and are shaded).

In the regular 4* k*-gon, there are *k* sets of mutually perpendicular pairs of opposite sides. Hence there must be at least *k* rectangles in the dissection.

Note that in the diagram we can also identify a rectangle (in fact three exist) based on the two other pairs of opposite sides.

## Competitions and the subject of mathematics itself

Teachers have various motivations for entering their students in competitions, and there are various motivations for mathematicians who elect to get involved with organisation of competitions and composing problems for use in competitions.

There would be scope for conducting a research project to quantify how much the various motivations might apply. Motivations for teachers can be to help talented students discover more mathematics, to help get an independent assessment of student abilities, sometimes providing useful surprises for teachers. Motivation for getting involved in organisation could involve helping to profile the importance of mathematics as a curriculum subject and improving student confidence with the extra experience.

Some teachers simply enjoy the experience of working with others in composing problems. Taylor and McAvaney (2020) wrote of their experience on the problems committee of an Australian competition, the Challenge, which is the subject of a paper by McAvaney in this special issue. They wrote of a number of cases where the work they and their colleagues had done had resulted in publications in refereed mathematics journals, thus contributing to the subject itself. They and colleagues had made such discoveries while considering problems which had been submitted to their committee for possible use, and also while attempting to design extension problems aimed at more interested and/or talented students.

The article by Taylor and McAvaney (2020) also noted that these papers arising from investigations by problems committee members sometimes found new methods for solving problems. An example was their introduction (McAvaney et al. 2012) of a graphical method to solve the so-called spouse-avoidance problems, in which one finds ways of organising mixed doubles tournaments in which players never partner their spouses.

Another new method they referred to is the method of the moving parallel, discussed immediately above, under the syllabus section.

These results often arise after problems committee members try to find multiple solutions independently of each other (one of the beauties of mathematics is that most problems can be solved in different ways, while always yielding the same result).

Another point noted in the Taylor and McAvaney paper is that some methods which had been lost in time have been revived, such as the revival of barycentric coordinates, as a particularly useful method in finding things like collinearities.

They also noted how some of the problems they had set resulted after being inspired by other results in the literature. Interesting theorems can be recontextualised into nice problems. In this special issue, He et al. (2022) illustrate this very point, for example describing a problem developed in China based on a theorem by Terence Tao. And the Taylor and McAvaney article describes a problem based on Erdős’ 1932 Discrepancy Problem recontextualised into an everyday situation.

Also in this special issue, similar experiences that are shared with colleagues in many other countries around the world can be found.

## Competitions and other areas of mathematics education

### Research

Competitions have also provided increased research capability in various aspects of mathematics education. In what follows we will examine a sample of what can be or has been done.

As an example, some research experiences have been enabled by the large inclusive competitions being on computer data bases; these include gender studies (see some intriguing results in Landim’s article (2022) on the Brazilian Olympiad for Public Schools which has an annual participation of some 18 million students).

In the past the main Australian competition had penalties for wrong answers which enabled risk study. Atkins et al (1991) were able to use the Ziller statistic to track risk taking attitudes by boys and girls of various ages.

Competitions form a major component of challenge in mathematics, with ICMI conducting its 16th Study on this topic, the final volume being edited by Barbeau and Taylor (2009).

Furthermore, activity, results and research pertaining to problem-solving competitions have the potential for strong interaction with other lines of research in mathematics education, both with regard to its practical objective of developing students’ mathematical thinking, as well as its theoretical aim of characterizing such thinking (Schoenfeld, 2000).

### Problem solving and posing

Emphasis on problem solving has been one of the main areas of research and practice in mathematics education for at least the last forty years. Recent trends have included problem posing alongside problem solving with especially noteworthy contributions coming from math educators such as Ed Silver and Jinfa Cai. For the first time the corresponding topic study group for ICME 14 includes problem posing alongside problem solving as areas of research and discussion. The activity and organization surrounding competitions is highly fertile on both fronts.

Problems can be created that require students to relate different fields of knowledge in new and enriching ways, cross-fertilizing mathematical topics, opening up opportunities for students to build networks of understanding, to deepen, widen and account for complexity in their conceptual images, as well as to develop autonomous and creative patterns of thought.

The wide range of problem-solving competitions offers almost every kind of problem imaginable concerning not only every topic of school mathematics, but also opening new topics and new interrelations among topics, as well as inviting new or unusual ways of analysing and thinking about them, thus enriching the school curriculum and pushing its boundaries (Sánchez and Nieto, 2022).

Although the main emphasis in research on problem posing concerns empowering students to pose problems of their own design, competitions provide examples of an ample range of problems that can be created touching on every topic of school mathematics and expanding the vision of how these can be looked on in new ways, going far beyond the textbooks and standardized tests and thus broadening the scope and sharpening the vision of the teacher (Cáceres et al. 2022).

Much can be learned from studying competition problems that squeeze the mathematics out of everyday situations or comparing problems from examinations such as PISA or TIMSS with those from problem-solving competitions and identifying the pretensions of each with regard to creating conditions for the student to develop his/her mathematical thinking.

Finally, comparison of problems from inclusive and more exclusive (national or regional) competitions shows an ample range of possibilities for the teacher as (s)he attempts to elicit the highest effort from each student.

### Constructing robust meaning

Competitions introduce interpretations and interrelations that contribute to the construction of robust meaning for mathematical concepts (robust concept images) frequently absent from the classroom.

We can illustrate this, for example, analysing a simple but classic problem from an inclusive competition such as the following one where the figure is shown and it is given that the length of the rectangle is twice the height, that E, F are midpoints of the respective sides and that the area of rectangle *ABCD* is 24 square centimetres. The question requires determining the area of the shaded region.
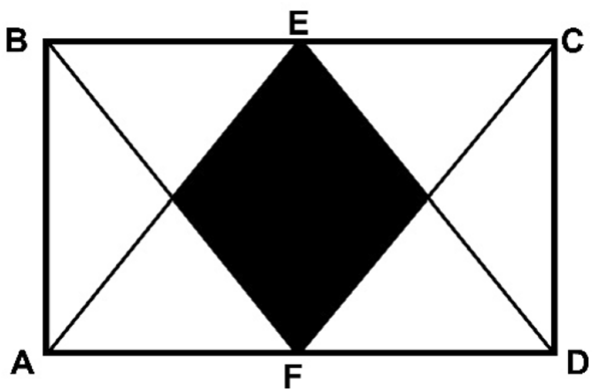


The formulation of the problem naturally suggests seeking how to decompose the figure in equal parts and calculating the area accordingly. This is, in fact, the way in which Euclid approached the question of area, emphasizing decomposition of planar regions and basing everything on the idea of figures (regions) of equal area, a singularly geometric viewpoint (Pérez, 2016). A competition problem such as the one posed revives this geometric approach providing insight into the meaning of the concept of area totally absent in the arithmetic (formula-based) approach so commonly encountered in school mathematics. In turn robust understanding thus empowers problem-solving or posing.

### Characterizing mathematical thinking

The raw material for analysing and characterizing mathematical thinking usually comes from the classroom experience or controlled experiments that eventually refer to school mathematics as the given environment. Thus, for example, the Van Hiele rubric for geometric thinking and Tall’s three-world structuring of mathematical thinking closely mirror the classroom (although perhaps not intentionally), and structure their examination of mathematical thinking in terms of the traditional curriculum, introducing predetermined limits into their characterizations.

Competitions transcend the traditional curriculum, not ignoring but enriching it, and build opportunities for mathematical thinking beyond the aims of the classroom. Participation in competitions also transcends the environment established by the individual school and/or teacher. Thus, the mathematical thinking evidenced by students participating in competitions offers a doubly wider scope to the researcher, this being especially true of competitions with ample participation (thousands or millions of participants) and those where students write full solutions to singular problems.

### Competitions and a popular mathematical theory

In another direction, claims can be found in the literature whose analysis can be fruitfully addressed by research done from the standpoint of competitions. In what follows we will relate our considerations to a few selected cases, noting that we do not pretend to cover any topic fully. Consider the following statement.

“… the mathematical way of thinking and working is based on transformations of semiotic representations into others” (Duval, 2017).

Is this what problem solvers actually do when solving problems? Studying the full solutions of hundreds or even thousands of students from many different schools or countries to the same well-designed problem can certainly shed light on this claim.

The problems posed in different mathematical competitions, even many of the most elementary problems, are designed to promote mathematical thinking and give young students the opportunity to actually do mathematics and appreciate how mathematical thinking can empower them.

Much mathematical work done in school is algorithmic, routine, or mechanical in nature, especially that concerned with the manipulation of symbolic expressions, such as algebraic equations or formulas, into equivalent expressions that can be interpreted to reveal the meaning sought after. But we find it necessary to doubt that such dreary words as treatment or processing within a semiotic register, as used by some who cite the work of Duval, could reflect the mathematical thinking that leads from one result to another apparently diverse one, such as the introduction and use of colouring arguments in combinatorics as seen in the following section.

#### Problem solving and semiotic registers

Some of the most ingenious solutions to problems come from the changing of semiotic registers, not in the rather routine sense of approaching quadratic equations from graphic, table or symbolic form, or putting a geometric situation from classic synthetic geometry into analytic form in order to solve it, but rather from noticing or constructing a link between a given problem and an interpretation in what would normally be seen as unrelated to the situation.

An example of this can involve colouring and invariants when the situation apparently involves addition and sums. A seventh-grade student, who had seen colouring arguments used in tiling problems, recently approached the following classic problem from this point of view.

A solitaire game starts with a circle divided into six sectors, each of which contains a number, as shown.
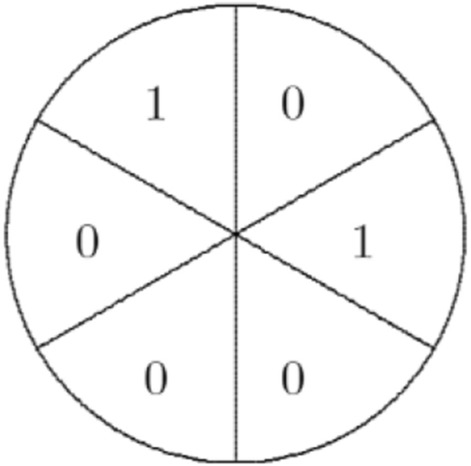


In each turn, 1 can be added to the numbers in each of two adjacent sectors. Show that it is impossible to make the numbers in all six sectors equal.

The student simply painted the sectors alternately as follows and argued that the sum of the numbers in the shaded sections is not equal to the sum of those in the unshaded sections, and that any move simply increases each of these sums by 1, so that, since they start out unequal, they will continue to be unequal, making it impossible for the numbers in all six sectors to be the same.
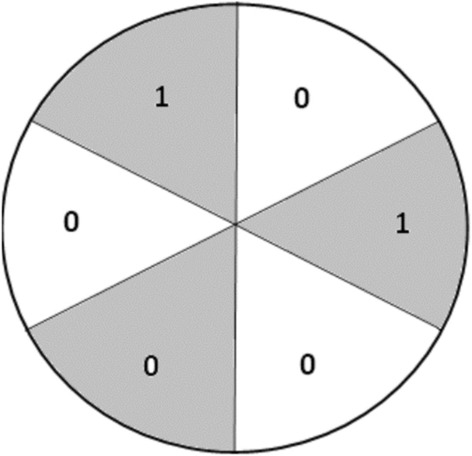


#### Processing and mathematics as a language

Practically any challenging problem requires a leap to something not obviously equivalent to what one has in hand as a starting point or given. Mathematical thinking cannot be reduced to representations and treatments because mathematics is not just a language to be learned, nor a means of representing certain givens, but rather a sort of elastic medium that permits not only the transition to nontrivial equivalents but also allows for the introduction of novel and unanticipated connections. In other words, mathematics is more than a language. Following Richard Feynman, we would say that mathematics is something like language plus logic.

Mathematics education must view mathematics in the same light if it is to empower students appropriately. It is more than a language, and thus the interpretations and applications of the theory of Duval which can are encountered in mathematics education as practiced on many different levels, where the symbolic formulation of a concept, problem or situation is seen as simply another semiotic register, are misleading. The notion of treatment within a register and specifically within the mathematical formulation fails to grasp that mathematics is more than a simple language (an alternative semiotic register) and hides its powerful nature, tending to put emphasis on manipulation and leading to a weakened conception of mathematical thinking.

The posing and solving of singular and challenging problems, as practiced in mathematics competitions, allows students to see and experience mathematics in a thoroughly enriching way, to grasp the power of mathematics in making unanticipated connections, and to develop their mathematical thinking as well as understanding. This experience leads far beyond the limits imposed in general by educational policy and unfortunately in many instances by the teacher in the classroom when following theories proposed in mathematics education.

### Competitions can shed light on the relationship between argument and proof

Much attention has been given to proof and proving in mathematics learning (Hanna & de Villiers, 2012). For example, preliminary studies of Olympiad papers show an interesting interplay between argument and proof.

Students’ written proofs in mathematics competitions frequently begin by describing what is going to be shown, and subsequently proceeding to show it. (Of course, this mirrors what many mathematicians do when introducing a proof, be it in class, in articles or in textbooks.) This is interesting in making a distinction between argument and proof when characterizing mathematical thinking, a distinction which is not always considered. It is also an important ingredient when assuming Hanna’s (Erdős’ and many others’) viewpoint that proof and proving in the classroom should address only those proofs that show why a statement is true, obviating proofs that only show that a statement is true. Specifying first the argument goes a long way to achieving this goal.

The ICMI Study on Proof and Proving edited by Gila Hanna and Michael de Villiers mentioned above contains a chapter on argumentation and proof in the mathematics classroom (Hanna et al., 2012, corrected edition 2021).

On the other hand, a rapid search of the internet produces statements such as this:

“A mathematical proof is an argument which convinces other people that something is true.” (https://math.berkeley.edu/~hutching/teach/proofs.pdf).

However, this glosses over an ongoing discussion in mathematics education circles as well as epistemological discussions about the nature of proof which have turned more urgent as the use of computational means to establish results becomes omnipresent.

The fact that young students spontaneously (i.e. without specific training) present their argument when solving a problem and subsequently develop the proof is evidence that there is a natural distinction between argument and proof that must be taken into account by researchers in mathematics education.

Our example concerns a seventh-grade student who had not previously received training and who in solving a certain problem exhibits a distinction between argument and proof.

The problem, of a type frequently seen in competition history and folklore, is the following.

Eight numbers are written around a circle. Each number is either 1 or − 1. A movement consists of changing the signs of one number and its two neighbours (that is, 1 becomes − 1 and − 1 becomes 1). Prove that from any initial configuration of these eight numbers, effecting this movement repeatedly, it is possible to obtain any other configuration of 1’s and − 1’s. For example, starting from the configuration 1, 1, 1, 1, 1, 1, 1, 1 around the circle it is possible to obtain − 1, − 1, 1, 1, 1, 1, 1, 1.

The student first presents his argument.

“In order to show that it is possible to make any combination of 1’s and -1’s that is wanted, it is easy to show that it is possible to change the sign of a single number without altering the signs of the other seven.”

Then he presents his proof of what he has just stated where he shows how to change the uppermost 1 to − 1 while leaving the other numbers invariant. He presents a series of movements following the arrows as shown.
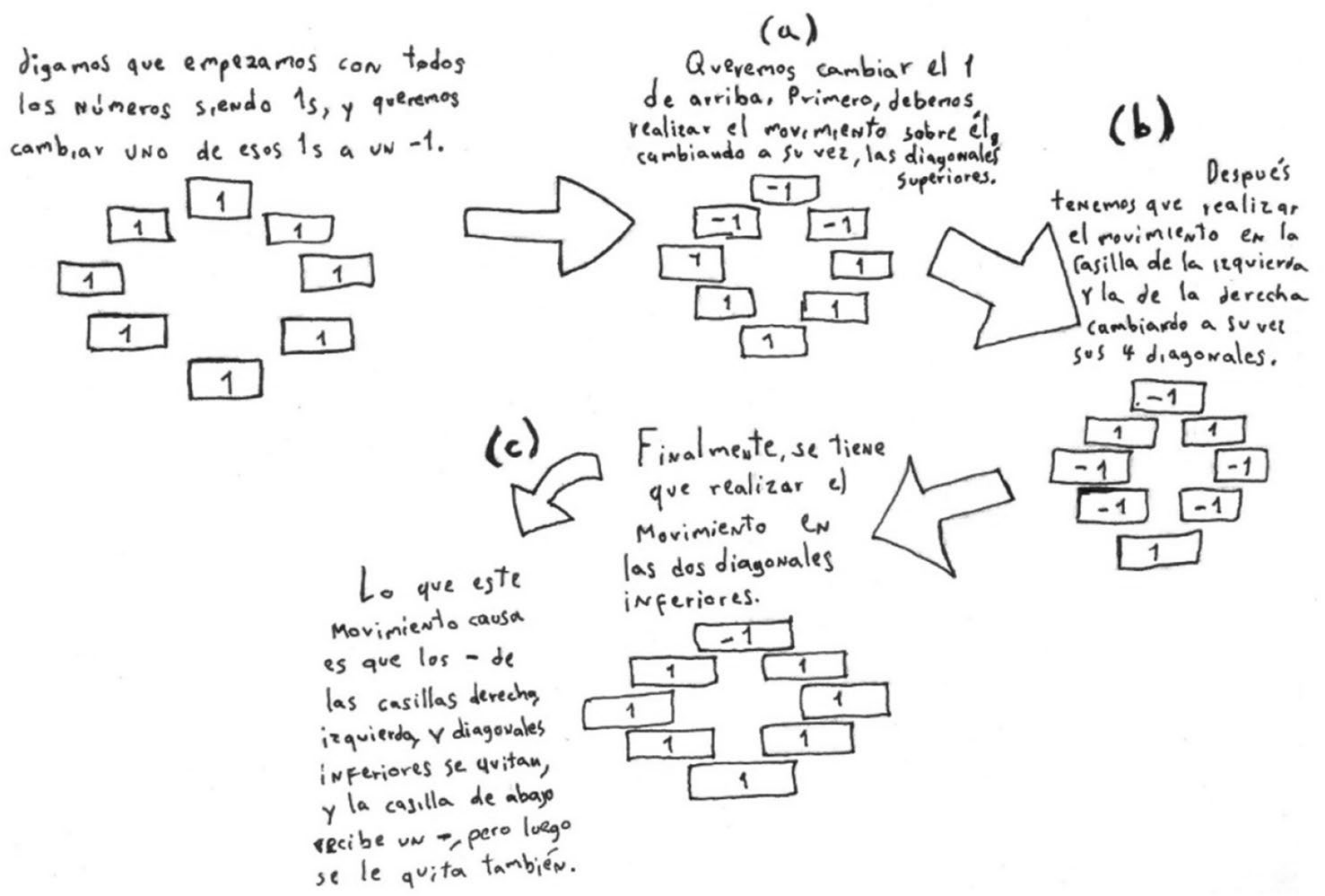


The text has been left deliberately in Spanish to show that the proof can be understood even when the text is not fully comprehended.

Finally, he analyses and comments on what he has done.

“This process substantiates and makes real the statement that it is possible to arrive at any desired combination of 1’s and − 1’s. Regardless of the position of the 1 or -1 that one wishes to change, it will always be possible to do so.”

There is no doubt here that the argument is proposed and then the proof is constructed, thus shedding light on the relationship between argument and proof especially given that they follow in quick succession in a problem-solving situation where time constraints do not permit extended periods of reflection that can result in their intermingling.

Furthermore, it seems that this can be related to the epistemological discussions revolving around computer-generated proofs. Does the mathematician and/or programmer, seeking to prove a theorem or arrive at a mathematical result, generate the argument? Does the computer “simply” substantiate the argument (generate the proof in the sense given above)?

Where can this be seen, for example, in topic study groups at ICME14, 2021? In TSG 16 titled “Reasoning, argumentation and proof in mathematics education”, we find that the first two among the issues considered of interest to this group are:

“Historical, philosophical, epistemological aspects of reasoning, argumentation and proof in mathematics and in mathematics education.

Theoretical and methodological approaches to examine epistemological, cognitive, and didactical issues in the teaching/learning of reasoning, argumentation and proof.” (https://www.icme14.org/static/en/news/37.html?v=1631685744414).

The group proposes that argumentation is more general than proof, being found in rhetoric and other fields different from mathematics.

It is especially true of so-called exclusive competitions which, by constantly proposing ever new problems that call for argumentation and proof as we have shown, expose a structure in mathematical thinking that can still be done without being obfuscated by the sophisticated mathematical language employed by experts, thus elucidating thought processes and relationships that may be interred in formal mathematical learning.

## Visualization and visual thinking

For well over fifty years there have been exchanges between mathematicians and mathematics educators looking at the role visualization plays in each of the two fields in relation to mathematical thinking (see for example Clements, 2014). There has also been important interaction between mathematics educators and professionals in the cognitive sciences concerning visualization in the mathematical learning process. It is therefore not surprising that a vibrant topic study group at ICME14 concerned itself with visualization and visual thinking. In its statement of purpose, TSG 23 says:

“In mathematics education research, visualization is generally referred to as the product and the process of creating, using, interpreting, and reflecting on visual information. It … is a vital component of conceptual understanding, reasoning, problem solving and proving. …” It goes on to state interest in

• “Visualization as a cognitive process. This would include visualization and reasoning, justification, argumentation, imagination, and difficulties with visualization.

• Visualization as a mathematical construct. This would include visualization and mathematizing, visualization and generalizing, visualization as a mathematical proof.” (https://www.icme14.org/static/en/news/37.html?v=1631685744414).

Mathematical problem-solving competitions have brought many aspects of visual thinking (as opposed to simple visualization) to the fore in a variety of contexts and in differing roles and explored new worlds of couching interesting and challenging mathematical problems in visual form. This is especially true of so-called inclusive competitions such as the Math Kangaroo. Consider the following problem.

Kadett (grades 7 and 8) 2019. Problem 13. Which of the tiles shown cannot be formed by combining the two given pieces?
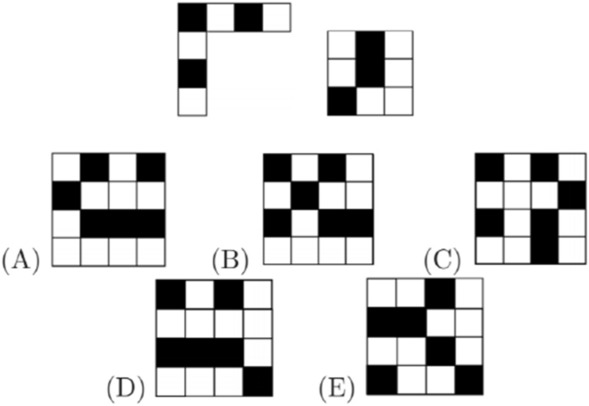


Given the time restraints, the student must rotate the pieces and fit them together using visualization (imagination) while if this were a homework assignment (s)he could make the pieces and manually rotate and fit them together. Thus, the problem invites visual thinking.

Kadett 2017 Problem 29. Sarah wants to write a positive whole number onto every tile in the number wall shown, so that every number is equal to the sum of the two numbers on the tiles that are directly below. What is the maximum number of odd numbers Sarah can write on the tiles?
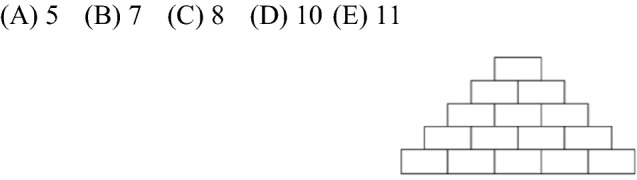


The figure comports and communicates visually ten interrelated mathematical statements that must be taken into account when solving the problem. Visualization here communicates mathematical conditions which would be very clumsy to state in words and which then must be elaborated and analysed using notions of parity. [Answer: D].

The following problem also communicates the “givens” of the situation visually but additionally the representations promote visual thinking as opposed to simple visualization of conditions given as in the previous case. For example, introducing a balance scale to represent an equation not only allows for visualizing the concept of equality, but permits operating visually on the equation allowing the student to find a solution to the question posed.

Three balance situations are shown with four kinds of toys (three equations with four variables are given). The question asks to balance one doll with a certain number of fish (express one variable in terms of another). Visual thinking, not simply visual representation, is involved as students imagine adding or removing figures in a way that will maintain a balance.
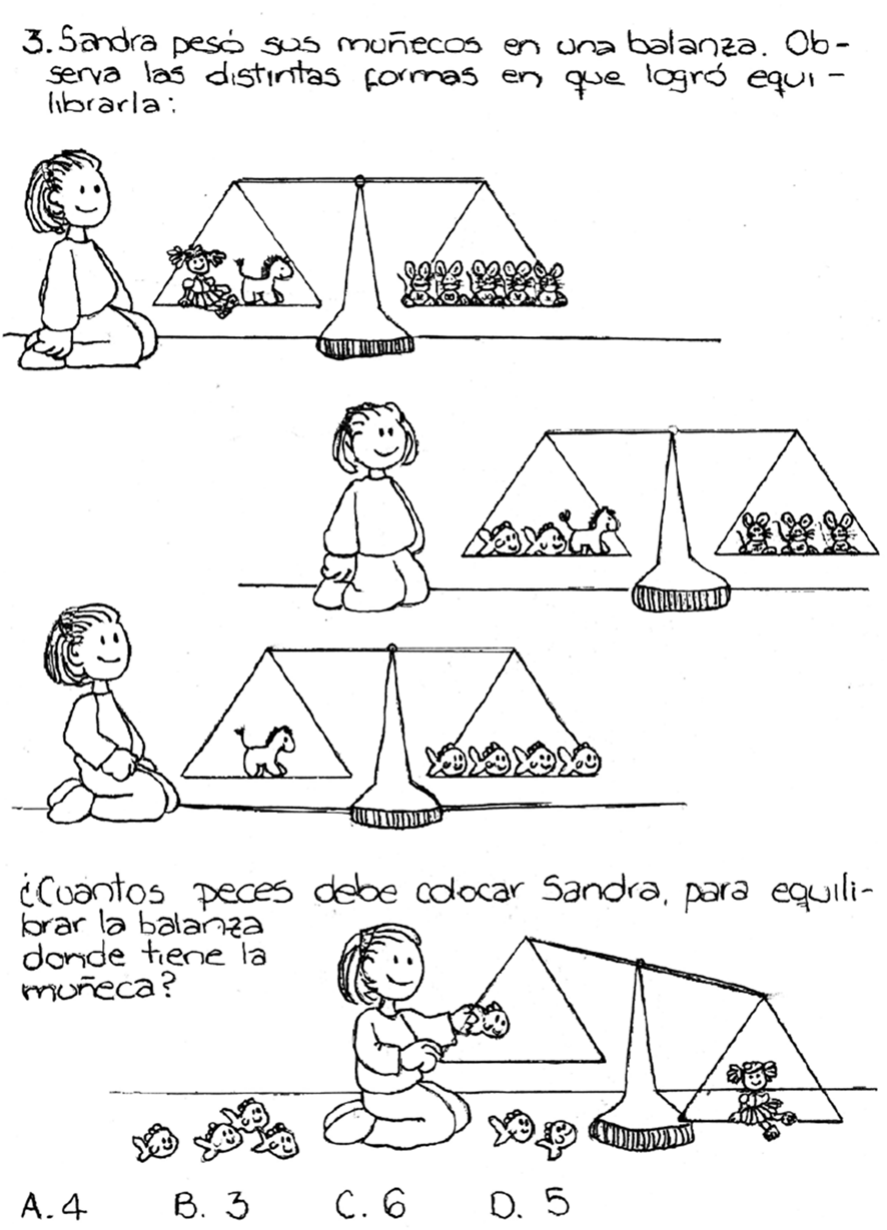


The written text states: “Sandra was weighing her toys on a balance scale. Observe the different ways in which she was able to get them to balance (obtain equilibrium)”. Then the question is asked: “How many fish should Sandra put on the scale to get them to balance with the doll?”.

It is to be acknowledged that the level or quality of visual thinking can be limited by time restrictions in all but long-term competitions such as the Challenge in Australia or math research camps such as the one oriented by the Tournament of Towns.

## Competitions and the popularization of mathematics

Although the popularization of mathematics as envisioned by the corresponding topic study group at ICME14 does not include a reference to problem-solving competitions, from the standpoint of many competitions there is a conscious and conscientious effort to contribute significantly to the popularization of mathematics. This is one of the central aims of the Math Kangaroo competition and can be appreciated in the problems created for it. It is widely accepted that primary school mathematics as generally experienced contributes little to the development of genuine mathematical thinking; problems from the Kangaroo illustrate how elements admitting mathematical thinking and the mathematics that supports many everyday experiences can show students more clearly what mathematics is and how mathematics can empower their thinking (Akveld et al. 2020).

## Range of topics in this special issue

While preparing this special issue on competitions, we have looked to convey an idea of the many branches of the field spanning the panorama of mathematical problem-solving competitions in the world today.

We study the history of competitions and especially recent innovations and trends. Special attention is given to the more recent history of the field (Kenderov, 2022).

International leadership and support for the community of those working in the field of competitions is the province of the World Federation of Mathematics Competitions (WFNMC) whose current president analyses the organization, its goals and achievements (Bankov, 2022). WFNMC is the international professional society for competition organisers, publishes its own journal, holds its own conferences, hosts its own website, confers awards for outstanding service to the field (Paul Erdős Award), and is an organization affiliated with ICMI.

A perspective of the history of mathematics competitions, dating back to 1894 in the modern era, their place as an integral part of the educational system, as well as recent technological trends in competition design, are given in the paper by Kenderov (2022).

Alexander Soifer shares a commentary on a particular mathematics competition founded on the initiative of a single person and its impact on the lives of its participants.

The paper by Geretschläger and Donner (2022) provides a look at competitions aimed at popularizing mathematics and giving support and materials to enrich the classroom, such as the Kangaroo competition that annually reaches some six million students in 88 countries (79 countries which are provisional or full members of the association and 9 countries currently in applicant status). This competition is present with two articles devoted to the creation of problems and the impact on teachers in different countries (Geretschläger & Donner, 2022; Cáceres et al. 2022).

Professors He, Ling and Xiong from China (2022) present an analysis of the competitions scene in that country and especially address the issue of the creation of original problems for the competitions as well as the relationship of competitions to career choices.

McAvaney (2022) presents an analysis of a competition that centres on long term solution of problems for young (middle school) students that allows them to get a feel for really doing mathematics. In the event he discusses, teachers enter students whom they consider to be talented and the students develop their classroom knowledge in stages to give them a broader perspective of the subject of mathematics.

We cover a range of competitions that have had significant impact on the development of young students, their educational communities, and their region of the world, and ask questions that point to future research with articles devoted to the International Mathematical Olympiad (IMO) (Saul & Vaderlind, 2022), the Iberoamerican Mathematics Olympiad (IbMO) (Fauring et al. 2022), and the Pan African Mathematics Olympiad (PAMO) and African competitions in more generality (Baker et al., 2022).

The possibility of competitions that address the problem of equity and inclusion, and directly benefit teachers, is illustrated by an article analysing aspects of the Brazilian Mathematics Olympiad for Public Schools that reaches some 18 million students each year (Landim, 2022).

In the paper by Cáceres et al (2022) a study is made of teachers in various countries covering a number of cultures, to determine their various views on the educational value of competitions.

Finally, Nieto and Sánchez (2022) discuss competition syllabi and their possible interaction with the modernization of school syllabi.

## Some conclusions and closing remarks

Each competition in its design reflects its educational and mathematical objectives, the population it intends to serve, its priorities and its dreams. The resulting structure is brought to life by the creation and selection of original problems conveying geniality, surprise, beauty and elegance. The articles contributed by McAvaney (2022), He et al. (2022) and Geretschläger and Donner (2022) connect directly with these components of design.

Competitions bring together practicing mathematicians, mathematics educators, teachers and students conforming a community with an especially rich cross section representing the shared interests and goals of these usually rather disparate groups.

While competitions are predominantly extracurricular, their relation to the curriculum, their pertinence for research related to other fields of mathematics education, their interaction with the field of mathematics itself reveal their role as an integral part of the educational process as argued by Kenderov (2022).

Differences between the trajectories of regional competitions such as the Iberoamerican (IbMO) and Pan African (PAMO) Olympiads illustrate the importance of varying factors, such as financial support and official sanctioning of competition activity at the regional and/or ministerial levels. At the same time, they reveal tensions that must be resolved within the community in order to assure initiatives that can prosper.

The emerging realities and the impact that competitions have on different scientific and educational shareholders are objects of study and research, as underlined by the articles contributed by Landim (2022) and by Cáceres et al. (2022), while those of Bankov (2022) and of Saul and Vaderlind (2022) look to future lines along which that research can develop.

Both explicit changes enacted and the more implicit evolution of the design of a competition reflect evaluation of results, reorientation of goals, variations in the participating population, or new external circumstances.

One of these, the ongoing coronavirus pandemic, has already changed the ways in which competitions are organized and realized, and most certainly will affect the future of the communities involved. How will “friendly” competition be replaced by virtual competition where there is no direct exchange between students, mentors or team leaders which has led in the past to international friendships and collaborations stretching far into the future?

More sentimentally, but of high importance to international understanding, no outings, excursions, chats in the cafeteria, late night get togethers or spontaneous football matches. No direct enriching experience of the culture, food, climate, natural environment, architecture of the host country, no listening to its language, its music and the sounds of its streets.

For it cannot be denied that competitions run virtually require significantly reduced funds and are almost certain to prevail. This is true for the organizers but also for the participants. Financial obstacles to participation will vanish almost entirely. In this new scenario, how will the seedlings of friendship and cooperation or future scientific collaborations fare? How will the enormous benefits of community survive, much less flourish?

## References

[CR1] Akveld, M., Caceres-Duque, L. F., & Geretschläger, R. (2020). Math Kangaroo. *Mathematics Competitions*, *33*(2), 48–66. Australian Maths Trust.

[CR2] Atkins WJ, Leder GC, O'Halloran PJ, Pollard GH, Taylor P (1991). Measuring risk taking. Educational Studies in Mathematics.

[CR3] Baker, L., James K., Labuschagne, P., Aloui, K., Weitbrecht, J., & Kariv, J. (2022). Mathematical Competitions in Africa: Their prevalence and relevance to students and teachers. *ZDM - Mathematics Education,**54*(5), in this issue. 10.1007/s11858-022-01347-5 PMC8988107 35411210

[CR4] Bankov, K. (2022). The Role and Significance of the World Federation of National Mathematics Competitions in the International Mathematics Education Community. *ZDM - Mathematics Education,**54*(5), in this issue.

[CR5] Barbeau, E. J. & Taylor, P. J. (Eds.). (2009). *Challenging mathematics in and beyond the classroom: The 16th ICMI study*. Springer Science & Business Media.

[CR6] Bollobás, B. (1997). Paul Erdős—life and work. In *The Mathematics of Paul Erdös I* (1–41). Springer. 10.1007/978-3-642-60408-9_1.

[CR7] Cáceres, L., Crawford, D. & Akveld, M. (2022). The impact of Maths competitions on teachers and their classrooms in Puerto Rico, Switzerland and the UK: A comparative study. *ZDM - Mathematics Education,**54*(5), in this issue.

[CR8] Clements MA (2014). Fifty Years of Thinking About Visualization and Visualizing in Mathematics Education: A Historical Overview. In Mathematics & Mathematics Education: Searching for Common Ground.

[CR9] Duval, R. (1999). *Representation, Vision and Visualization: Cognitive Functions in Mathematical Thinking. Basic Issues for Learning.* In Proceedings of the Annual Meeting of the North American Chapter of the International Group for the Psychology of Mathematics Education, Cuernavaca, Mexico.

[CR10] Duval R (2006). A cognitive analysis of problems of comprehension in a learning of mathematics. Educational Studies in Mathematics.

[CR11] Duval, R. (2017). *Understanding the mathematical way of thinking-The registers of semiotic representations*. Springer International Publishing.

[CR12] Engel, W. (2009). *The German Teams at the International Mathematical Olympiads 1959–2008*. Bock.

[CR13] Falk de Losada M (2012). Corrientes del Pensamiento Matemático del Siglo XX, Volumen 1.

[CR14] Falk de Losada, M. (2017). Are mathematics competitions changing the mathematics that is being done and the way mathematics is done? In *Competitions for Young Mathematicians*, 329–350. Springer.

[CR15] Falk de Losada, M. F. (2020). The Impact of Mathematical Olympiads on the Mathematics Community of Colombia. *Engaging Young Students in Mathematics through Competitions—World Perspectives and Practices: Volume II: Mathematics Competitions and how they relate to Research, Teaching and Motivation,* 139–159. World Scientific.

[CR16] Fauring, P., M. Gaspar & M. Losada (2022). The Iberoamerican Mathematics Olympiad: Competition and Community. *ZDM - Mathematics Education,**54*(5), in this issue.

[CR17] Freudenthal H (1969). ICMI report on mathematical contests in secondary education (olympiads) I. Educational Studies in Mathematics.

[CR18] Galvin F (1990). Letter to Editor. American Mathematical Monthly.

[CR19] Geretschläger, R. & Donner, L. (2022). Writing and Choosing Problems for a Popular High School Mathematics Competition. *ZDM - Mathematics Education*, 54(5), in this issue.

[CR20] Gowers, W. T. (2000). The two cultures of mathematics. *Mathematics: Frontiers and Perspectives*, AMS*,* 65–78.

[CR21] Gowers, T., Barrow-Green, J. & Leader, I. (Eds.). (2008). *The Princeton companion to mathematics*. Princeton University Press.

[CR22] Hanna, G. & de Villiers, M. (2012). Aspects of proof in mathematics education. *Proof and proving in mathematics education: The ICMI Study*, 1–10. Corrected edition 2021.

[CR23] He, Y., Ling, T. & Xiong, B. (2022), Mathematics competitions in China: practice and influence. *ZDM—Mathematics Education,**54*(5), in this issue.

[CR24] Kasimatis EA (1989). Dissections of regular polygons into triangles of equal areas. Discrete & Computational Geometry.

[CR25] Kenderov, P. (2022). Mathematics Competitions – an Integral Part of the Educational Process. *ZDM - Mathematics Education,**54*(5), in this issue. 10.1007/s11858-022-01348-4 PMC8970973 35382411

[CR26] Kline, M. (1972). *Mathematical Thought from Ancient to Modern Times.* Oxford University Press, 621–22.

[CR27] Landim, C. (2022). The Brazilian Public Schools Math Olympiad (OBMEP), 15 Years Promoting Social Mobility through Academic Achievement. *ZDM - Mathematics Education,**54*(5), in this issue.

[CR28] Lesh, R. & Sriraman, B. (2010). Re-conceptualizing mathematics education as a design science. In *Theories of mathematics education*, 123–146. Springer.

[CR29] McAvaney, K. (2022). Mathematics challenge for young Australians. *ZDM - Mathematics Education,**54*(5), in this issue.

[CR30] McAvaney K, Taylor P, Thornton S (2012). Euler and the mathematics challenge for young Australians. Gazette of the Australian Mathematical Society.

[CR31] Nieto, J. & Sanchez, R. (2022). The Curriculum for Mathematics Competitions. *ZDM - Mathematics Education,**54*(5), in this issue.

[CR32] Pérez D, Falk de Losada M (2016). Cuestionando las orientaciones del currículo: Un enfoque alterno para el concepto de área. VIDYA.

[CR33] Prediger S, Gravemeijer K, Confrey J (2015). Design research with a focus on learning processes: An overview on achievements and challenges. ZDM Mathematics Education.

[CR34] Reid C (1970). Hilbert.

[CR35] Saul, M. & Vaderlind, P. (2022). The International Mathematical Olympiad, Age 62: What we know and What we would like to know. *ZDM - Mathematics Education,**54*(5), in this issue.

[CR36] Schoenfeld AH (2000). Purposes and methods of research in mathematics education. Notices of the AMS.

[CR37] Soifer, A. (2022). The Soifer (formerly Colorado) Mathematical Olympiad, why it was founded, bridge between its problems and mathematics, and lives of its winners: An essay. *ZDM - Mathematics Education,**54*(5), in this issue 10.1007/s11858-021-01320-8 PMC8720553 35003375

[CR38] Stylianides AL (2007). Proof and proving in school mathematics. Journal for Research in Mathematics Education.

[CR39] Taylor, P. (2013). *Former Australian Mathematics Olympians*. https://sites.google.com/site/pjt154/home/a2-imo-students.

[CR40] Taylor P (2015). Classifying methods of problem solving—And my favourites. Mathematics Competitions.

[CR41] Taylor, P. (2017). Chapter 12 Future Directions of Research in Mathematics Competitions, Soifer, Alexander (editor), *Competitions for young mathematicians: Perspectives from five continents. ICME-13 Monographs, Springer*.

[CR42] Taylor, P. & McAvaney, K. (2020). Examples of Mathematics and Competitions influencing each other. In *Engaging Young Students in Mathematics through Competitions—World Perspectives and Practices: Volume II: Mathematics Competitions and how they relate to Research, Teaching and Motivation,* 15–33. World scientific.

[CR43] XI Competición Interuniversitaria Iberoamericana de Matemáticas (2019). Problems and solutions. Unpublished. Guanajuato, Mexico.

